# Electrospinning of Essential Oils

**DOI:** 10.3390/polym12040908

**Published:** 2020-04-14

**Authors:** Elisa Mele

**Affiliations:** Materials Department, Loughborough University, Epinal Way, Loughborough LE11 3TU, UK; e.mele2@lboro.ac.uk

**Keywords:** electrospinning, essential oils, nanofibres, terpenoids, phenylpropanoids

## Abstract

The extensive and sometimes unregulated use of synthetic chemicals, such as drugs, preservatives, and pesticides, is posing big threats to global health, the environment, and food security. This has stimulated the research of new strategies to deal with bacterial infections in animals and humans and to eradicate pests. Plant extracts, particularly essential oils, have recently emerged as valid alternatives to synthetic drugs, due to their properties which include antibacterial, antifungal, anti-inflammatory, antioxidant, and insecticidal activity. This review discusses the current research on the use of electrospinning to encapsulate essential oils into polymeric nanofibres and achieve controlled release of these bioactive compounds, while protecting them from degradation. The works here analysed demonstrate that the electrospinning process is an effective strategy to preserve the properties of essential oils and create bioactive membranes for biomedical, pharmaceutical, and food packaging applications.

## 1. Introduction

Synthetic drugs, preservatives and pesticides used in pharmaceutics, cosmetics, medicine, the food industry and agriculture are a source of health and environmental concerns worldwide [1,2]. The unregulated and extensive consumption of antibiotics to treat human and animal infections and stimulate the growth of food-producing animals (cattle, pigs, and poultry) has resulted in the emergence of antibiotic resistance in pathogens. The rate of hospitalisation and mortality of patients infected with drug-resistant microorganisms has increased, posing major health threats. Lipophilic pesticides, such as organochlorines (OCs), although banned in many countries, are still being applied to eradicate pests [3,4,5]. They are hazardous organic pollutants that persist in the environment, bioaccumulate in terrestrial and aquatic ecosystems, and enter the food chain. The exposure to OCs has been linked to acute and chronic diseases in humans, such as type-2 diabetes, Parkinson, and cancer. The potentially disastrous consequences of synthetic chemicals on the global population and the environment have motivated the search for less toxic and more environmentally friendly alternatives. This has seen a renewed interest toward natural products and especially essential oils (EOs) for their antibacterial, antifungal, antiviral, antioxidant, anti-inflammatory, and insecticidal activity [6,7,8,9].

Essential oils are complex mixtures of volatile compounds that are synthesised by plants for defence and signalling purposes [10,11,12,13]. EOs protect plants against herbivores and harmful insects by inhibiting their appetite and attracting their natural opponents. They repel pathogenic microorganisms by targeting their nervous, digestive or endocrine systems, and reducing their growth. EOs promote plant adaptation to abiotic stresses, such as temperature, draught, light, carbon dioxide, and ozone levels, by protecting the photo synthetic apparatus and increasing thermal tolerance of photosynthesis. In addition, they play a crucial role in attracting beneficial animals, such as species-specific pollinators and seed dispersers, to facilitate plant reproduction.

**Figure 1:**
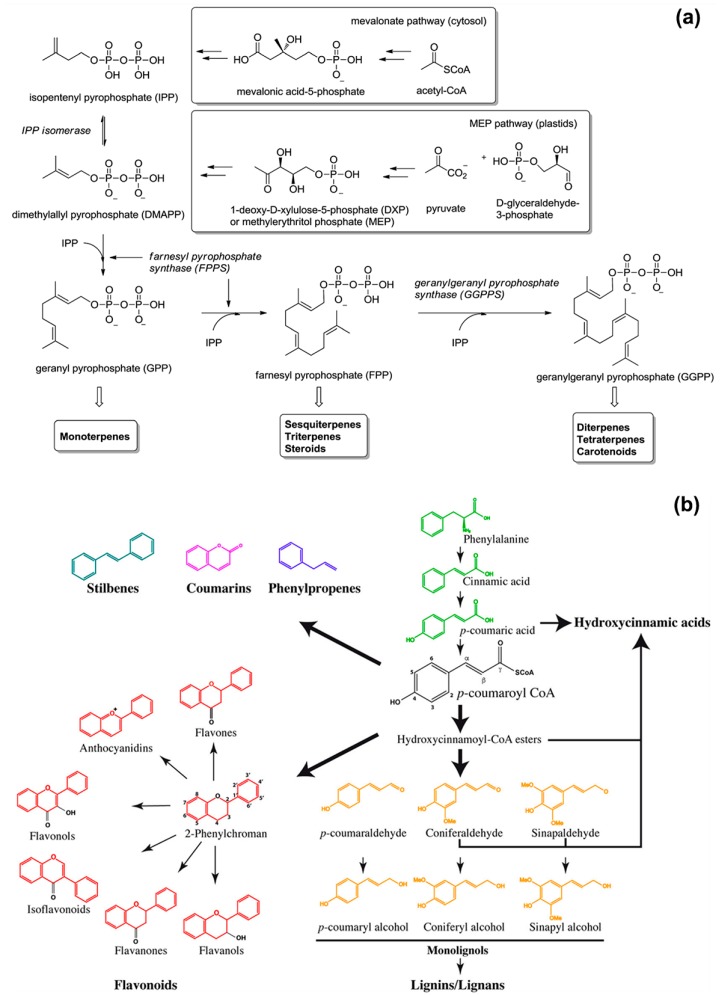
Schematic representations of the biosynthesis of: (**a**) isoprenoids, reproduced from Ref. [20] with permission from the Royal Society of Chemistry; (**b**) phenylpropanoids, reproduced from Ref. [21].

The functionality and scent of EOs derive from their main chemical components that belong to the class of isoprenoids and phenylpropanoids [10,12,14,15]. Isoprenoids, also known as terpenoids, are the predominant constituents of EOs and derive from 5-carbon precursors (Figure 1a): isopentenyl pyrophosphate (IPP) and its isomer dimethylallyl pyrophosphate (DMAPP). Higher plants synthesise IPP and DMAPP via two independent pathways: the mevalonic acid (MVA) pathway in the cytosol, and the methylerythritol phosphate (MEP) pathway in the chloroplasts (organelles used for the photosynthesis) [16,17]. Prenyltransferase enzymes catalyse the condensation (head-to-tail) of one IPP molecule with one DMAPP molecule to yield geranyl diphosphate (GPP, C_10_), which further condensates with another IPP molecule to form farnesyl diphosphate (FPP, C_15_). The addition of another IPP molecule to FPP gives geranylgeranyl diphosphate (GGPP, C_20_). Then, monoterpene synthases, sesquiterpene synthases, and diterpene synthases convert GPP to monoterpenes (C_10_), FPP to sesquiterpenes (C_15_), and GGPP to diterpenes (C_20_), respectively. Well-known representatives of terpenes are: limonene (found in citrus oils), thymol (found in thyme oil), and menthol (found in mint oil) for monoterpenes; β-caryophyllene (found in clove oil), chamazulene (found in chamomile oil), and β-nerolidol (found in jasmine oil) for sesquiterpenes [18].

Phenylpropanoids are secondary plant metabolites synthetized by the amino acid phenylalanine (primary metabolite) via the general phenylpropanoid pathway (Figure 1b) [19,20,21,22]. Their biosynthesis starts with the formation of cinnamic acid from phenylalanine by the action of phenylalanine ammonia-lyase (PAL). In the second enzymatic step, cinnamate 4-hydroxylase (C4H) catalyses the hydroxylation of cinnamic acid to *p*-coumaric acid, which is converted into coumaroyl-coenzyme A (coumaroyl-CoA) by 4-coumaroyl CoA ligase (4CL). Coumaroyl-CoA is the intermediate for the generation of various phenylpropanoid compounds, such as eugenol, myristicin, methyl cinnamate, chavicol, methyl chavicol, and estragole [11,23].

The chemical components of essential oils are volatile and susceptible to easy degradation due to temperature, light, oxygen, and moisture. Hence, encapsulating systems, such as micro- and nano-particles, capsules, droplets, cyclodextrin complexes, and fibres, are required to deliver still-functional EOs to a specific target and in a controlled fashion [24,25,26]. This review analyses the current state of the art on the release of EOs from polymeric fibres produced by electrospinning. After giving an overview of the electrospinning process, the review discusses in detail the research conducted so far on the incorporation of essential oils of cinnamon, oregano, peppermint, clove, thyme, and lavender in polymeric electrospun fibres. These EOs have been selected because the properties of the resulting fibres have been widely discussed in the literature.

## 2. Production of Polymeric Fibres by Solution Electrospinning

Electrospinning is a nanofabrication technique that operates at high electric voltages to extrude polymeric fibres with a diameter down to the nanometre [27,28,29,30]. Two main electrospinning approaches are available: solution electrospinning when the starting polymer is in solution with an appropriate solvent or solvent mixture; melt electrospinning when the polymer is processed in the molten state and no solvents are utilised. This review focuses on solution electrospinning, hereinafter referred to simply as electrospinning.

A typical electrospinning apparatus takes the form of a high-voltage power supply, a spinneret (usually a metallic needle) attached to a syringe, a syringe pump, and a conductive collector (usually copper or aluminium) [27,31]. The first step of the electrospinning process consists in generating a pendant droplet of the polymer solution at the tip of the spinneret by pumping the liquid at a constant flow rate using the syringe pump. Then, an electric potential difference is generated between the spinneret, which is connected to the high voltage power supply, and the collector (usually grounded). With voltage increase, electric charges accumulate on the surface of the droplet till a critical voltage is reached (more than tens of kV). At this point, the electrostatic repulsion overcomes the surface tension and the viscoelastic forces of the polymer solution, and the droplet deforms into a conical shape, known as a Taylor cone. An electrified liquid jet is ejected from the apex of the Taylor cone and it is accelerated toward the collector. The jet moves in a nearly straight line in a region close to the spinneret tip (near-field region); it stretches, gradually decreases in diameter, and experiences bending or whipping instabilities away from the spinneret and in close proximity to the collector (far-field region). The instabilities, due to electrostatic repulsion of charges, determine further jet thinning and solvent evaporation with consequent jet solidification. Finally, solid fibres are deposited on the collector to form a non-woven mat.

The fabrication of defect-free electrospun fibres with the desired size and structure is achievable by a fine optimisation of all process parameters [32,33]. This includes control over the properties of the polymer solution (viscosity, molecular weight of the polymer, conductivity and boiling point of the solvent system, presence of additives), the applied voltage, the flow rate, the spinneret-collector distance, the environmental conditions (temperature and humidity), and the collector geometry. For example, solvent volatility is instrumental to engineer in-fibre porosity and generate fibres with high specific surface area [2,34,35,36]. Binary and ternary polymer solutions prepared with an appropriate selection of solvents and non-solvents (low boiling point solvents) have been investigated to induce phase separation events prior or during the electrospinning process and hence generate pores. In addition, the porosity of the overall electrospun mat (fibre packing density) can be adjusted by acting on the collector geometry, which in turn affects the external electric field and, consequently, the orientation and alignment of the fibres [27,37,38]. While deposition of randomly oriented fibres occurs on a planar, static collector, the uniaxial alignment of fibres can be obtained by a rotating mandrel, a pair of electrodes separated by air gap, or two parallel permanent magnets.

## 3. Electrospun Fibres Containing EOs and Their Applications

So far a wide variety of essential oils have been electrospun, including cinnamon [39,40,41,42,43,44], oregano [45,46,47,48], peppermint [44,49,50,51,52], clove [41,53,54,55,56,57], thyme [58,59,60,61], lavender [62,63,64], eucalyptus [65], ginger [66], tea tree [67,68], Manuka [68], black pepper [69], and sage [69]. This review focuses on works that report the addition of EOs to polymeric solutions before conducting the electrospinning process. Although studies on the electrospinning of specific chemical constituents of essential oils are available in the literature, these are not reported in this review.

### 3.1. Cinnamon Essential Oil

Cinnamon EO is extracted from the leaves and bark of ever green aromatic trees of the *Cinnamomum* genus and *Lauraceae* family, and distributed in China, India, America, and Australia [70]. Its main chemical component is cinnamaldehyde, which is active against pathogens such as *Escherichia coli* (*E. coli*), *Staphylococcus aureus* (*S. aureus*), *Porphyromonas gingivalis* (*P. gingivalis*), *Listeria monocytogenes* (*L. monocytogenes*), and *Bacillus cereus* (*B. cereus*) [71,72,73,74]. It has been demonstrated that cinnamon EO induces permanent damages to the morphology and permeability of the cytoplasmic membrane of *E. coli* and *S. aureus*, when the bacteria are exposed to EO doses in the range of 1–4 mg/mL [71,72]. The disruption of the membrane integrity causes leakage of nucleic acids and proteins, with consequent bacteria death. This phenomenon is more evident for *S. aureus* (Gram-positive bacterium), whose membrane is less resistant to hydrophobic molecules (such as EOs), than for *E. coli* (Gram-negative bacterium). Increased cell membrane permeability, loss of DNA, RNA and proteins, deformation and rupture of cells have been recorded also for *P. gingivalis* (a Gram-negative anaerobic bacterium responsible for chronic periodontitis) treated with cinnamon EO (dose of 6.25 μg/mL) [73].

**Figure 2:**
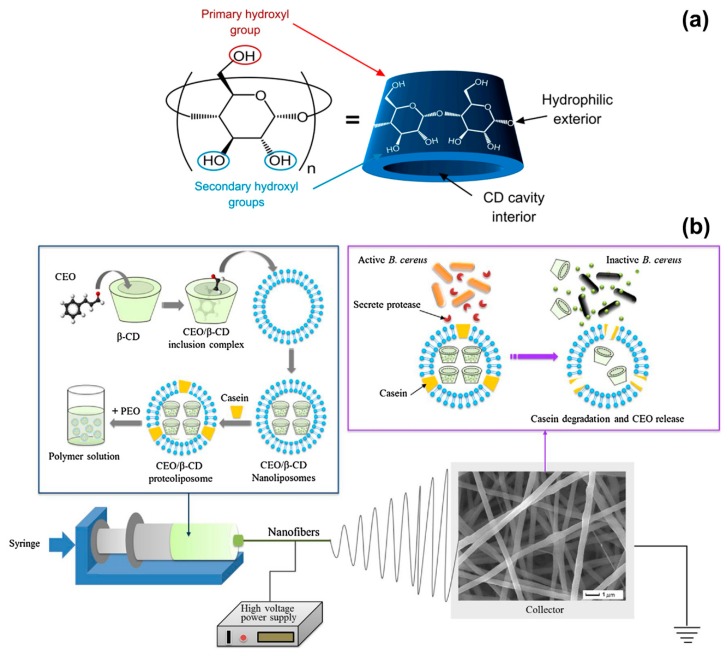
(**a**) Schematic representation of the structure of a cyclodextrin molecule, with indication of the hydrophilic external surface and hydrophobic cavity. Adapted from Ref. [76]. (**b**) Illustration of the formation of cinnamon EO/β-CD proteoliposomes contained in poly(ethylene oxide) (PEO) nanofibers, and release of the essential oil (EO) when the fibres are in contact with *B. cereus*. Reproduced from Ref. [43] with permission from Elsevier.

Cinnamon EO has been electrospun in combination with a range of polymers, including polyvinyl alcohol (PVA) [39,40], alginate/PVA [41], polylactic acid (PLA) [42], poly(ethylene oxide) (PEO) [43], and cellulose acetate [44], and the resulting fibres have been applied to food and biomedical sectors. In most works, complexes of cinnamon EO and cyclodextrins were processed. Cyclodextrins are natural cyclic oligosaccharides with a truncated cone shape characterised by a hydrophilic external surface and a hydrophobic interior cavity (Figure 2a) [26,75,76]. They are extensively used to form inclusion complexes with essential oils, which are trapped in the hydrophobic cavity, in order to improve EOs bioavailability and stability.

Antimicrobial and biodegradable packaging materials have been produced by incorporating cinnamon EO and β-cyclodextrin (β-CD) into PVA [39], PLA [42], and PEO [43] nanofibers. P. Wen and collaborators reported on PVA solutions prepared by dissolving PVA in water at a 6% w/w concentration and adding 2% w/w of cinnamon EO and 2% w/w of β-CD [39]. The electrospinning process was conducted with a voltage of 15 kV, a flow rate of 0.5 mL/h and a needle-collector distance of 15 cm to achieve an average fibre diameter of (300 ± 60) nm. The antimicrobial activity of cinnamon EO was tested against *E. coli* and *S. aureus*. The electrospun PVA/EO/β-CD mats generated inhibition zones of (28.9 ± 0.3) mm for *E. coli* and (30.5 ± 0.4) mm for *S. aureus*, and were slightly more effective than 50 μg/disk of the antibiotic kanamycin sulphate [(28.2 ± 0.2) mm for *E. coli* and (24.1 ± 0.5) mm for *S. aureus*]. MIC values (minimum concentration of fibres to inhibit the microorganisms growth) of 1.0 mg/mL for *E. coli* and 0.9 mg/mL for *S. aureus* were recorded, corresponding to 9–10 μg/mL of cinnamon EO (4–5 μg/mL of kanamycin sulphate were needed to achieve the same effect); whereas, MBC values (lowest concentration of fibres to kill 99.99% of microorganisms after 24 h of incubation) were of 8.0 mg/mL for *E. coli* and 7.0 mg/mL for *S. aureus*, corresponding to 70–80 μg/mL of cinnamon EO (effective as 15–20 μg/mL of kanamycin sulphate). Cinnamon EO not only stopped the growth of microorganisms but also increased the shelf-life of packed strawberries. When the electrospun PVA/EO/β-CD mats were applied as food packaging material (at 4 °C), the firmness of the fruit (one indicators of freshness) decreased by only 14% after 18 days of storage; while a decrease of 35% was recorded when a commercial fresh-keeping film was used instead of the electrospun mat. The efficacy of cinnamon EO, released from electrospun fibres, was demonstrated also for meat preservation, by the same group of authors [42]. In this case, PLA fibres containing cinnamon EO and β-CD inclusion complex (β-CD-IC) were electrospun from a solvent mixture of dichloromethane (DCM) and dimethylformamide (DMF, 3:1 DCM:DMF v/v ratio). PLA and EO/β-CD-IC concentrations were both of 10% w/v; while the EO:β-CD ratio was fixed at 1:9 to achieve an EO loading content of 10.8% w/w after complexation. The PLA/EO/β-CD-IC fibres produced had an average diameter of (850 ± 120) nm (voltage of 15 kV, flow rate of 2.5 mL/h and needle-collector distance of 15 cm) and exhibited MIC and MBC values of 1 mg/mL and 7 mg/mL for *E. coli* and *S. aureus*, respectively. The antimicrobial fibres prolonged the shelf-life of pork meat stored at 25 °C up to 8 days; while pork packed with fresh-keeping films decayed after 3 days. The results of these two works show that electrospun fibres are promising systems for the encapsulation and delivery of cinnamon EO to create active food packaging materials that are biodegradable and can delay food spoilage by inhibiting both Gram-positive and Gram-negative bacteria.

Innovation in the food packaging industry has recently seen the emergence of intelligent technologies based on compounds that not only prolong the shelf-life of food but more importantly respond, in a controlled and specialised fashion, to the presence of microorganisms [77]. In this scenario, L. Lin and collaborators have developed active PEO electrospun fibres that release cinnamon EO in the presence of *B. cereus*, which is one of the most frequent causes of food poisoning outbreaks [43,78]. Cinnamon EO was encapsulated in proteoliposomes, which are lipid nanovesicles incorporating membrane proteins and able to release the contained antibacterial agent only when they enter in contact with the target bacterium. Cinnamon EO was first complexed with β-CDs to enhance its stability and increase the encapsulation efficiency in proteoliposomes (Figure 2b). Solutions of PEO in water (5% w/v concentration) were prepared and mixed with cinnamon EO/β-CD proteoliposomes having an average size of (349 ± 36) nm. The electrospinning process (voltage of 25 kV, flow rate of 0.6 mL/hour and needle-collector distance of 12 cm) resulted in fibres with a diameter between 500 and 650 nm. When the fibres were exposed to culture medium containing *B. cereus* colonies, 80% of cinnamon EO was releases after 48 h at 37 °C, due to the activation of the proteoliposomes by the protease secreted by the bacteria (Figure 2b). A release of only 30% was instead detected without *B. cereus* under the same in vitro conditions. The suitability of the fibres as packing material was demonstrated on fresh beef samples that were inoculated with *B. cereus* and stored for 4 days at 25 and 37 °C. The fibres induced 99.99% reduction in *B. cereus* population and had a negligible impact on the sensorial quality of beef (limited variations in beef colour and texture). The outcomes of this work highlight the potential of electrospun fibres as engineered systems for the controlled release of natural antimicrobial compounds in the field of food preservation.

### 3.2. Oregano Essential Oil

Recent studies have reported on electrospun fibres containing oregano essential oils, extracted from *Origanum vulgare* [45,46,47] and *Origanum minutiflorum* [48], which are plants belonging to the *Lamiaceae* family and predominantly distributed in the Mediterranean, Euro-Siberian and Iran-Siberian areas. The major constituents of oregano EO are carvacrol and thymol, which have inhibitory effect on diverse microorganisms, including Methicillin-resistant *S. aureus* (MRSA), *E. coli, Bacillus subtilis* (*B. subtilis*), and *Saccharomyces cerevisiae* [79,80,81]. As other EOs, oregano EO acts on the bacteria cell membrane by disrupting its functions, inducing loss of cytosolic material and leakage of potassium ions, with eventual cell necrosis. Oregano EO has been proposed to inactivate biofilms, which are sessile colonies of bacterial cells strongly adherent to surfaces and poorly permeable to antibacterial agents and antibiotics [82]. Concentrations of this EO in the range of 0.3–1.0 mg/mL have been proved effective in completely eradicating 24-h old biofilms of *Acinetobacter baumannii*, *Pseudomonas aeruginosa* (*P. aeruginosa*) and MRSA within 1 h. Oregano EO permeated the biofilms and led to physical and morphological damages to the bacteria organisation. Oregano EO has been embedded in biodegradable fibres of chitosan and poly(caprolactone) (PCL) that have been electrospun from formic acid/acetic acid (1:1 volume ratio) solutions, using a voltage of 18 kV, a flow rate of 0.1 mL/h and a needle-collector distance of 15 cm [46]. The resulting mats (210–320 nm average fibre diameter), containing 1%, 3%, and 5% w/w of EO, induced a decrease of bacteria population in the range of 40–53% after 3 h of incubation. They were tested against four pathogens relevant to food packaging: *S. aureus*, *L. monocytogenes*, *E. coli* and *Salmonella enteritidis* (*S. enteritidis*). The release of the EO was investigated in-vitro in a buffer solution at 37 °C and pH 7.4. A burst release was recorded in the first 12 h, with values of ~10% for the lowest EO concentration, and ~32% for the highest concentration. This was attributed to oil present on the surface of the chitosan/PCL fibres. Then, the release gradually increased till reaching a plateau after 48 h, with values of 15–45% after 96 h, due to oil entrapped within the fibre volume. A similar biphasic release profile has been reported for poly (L-lactic acid-*co*-caprolactone)/silk fibroin (PLCL/SF) fibres that were electrospun from 1,1,1,3,3,3-hexafluoro-2-isopropanol (HFIP) solutions containing 2.5%, 5.0%, and 7.5% v/v of oregano EO [47]. The fibrous mats had an average fibre diameter of ~500 nm obtained by electrospinning at a voltage of 12–15 kV, a flow rate of 1 mL/hour and a needle-collector distance of 12–14 cm. They showed an initial burst release in vitro in the first 3 h (77% oil released from fibres with 5% v/v EO) and a steady release after 48 h (89% oil released from fibres with 5% v/v EO). The antioxidant and antitumor activity of the encapsulated oregano EO was investigated, instead of the antibacterial properties. The electrospun samples with 5.0% and 7.5% v/v of oregano EO exhibited the ability of scavenging 2,2-diphenyl-1-picryl-hydrazyl-hydrate (DPPH) free radicals in 30 min, with values (~90%) comparable to those of pure ascorbic acid (standard antioxidant agent) and higher than those of pure, non-encapsulated oregano EO (~65%). The excellent DPPH radical scavenging activity indicates that the high surface-to-volume ratio of the nanofibres facilitates the release of the antioxidant agent and enhances its therapeutic potential. In addition, the fibres with 5.0% and 7.5% v/v of oregano EO dramatically decreased the proliferation of 4T1 mammary carcinoma cells, achieving a complete antiproliferative activity at 72 h. Other studies have reported on the cytotoxicity of oregano extracts on tumour cell lines, and attributed it to some of its chemical constituents, such as rosmarinic acid and 4-terpineol, and their synergistic interaction [80].

The current literature on the electrospinning of oregano EO demonstrates that a sustained release of the bioactive agent can be achieved from polymeric nanofibres, and systems with a prolonged antibacterial, antioxidant, and antitumor activity can be developed.

### 3.3. Peppermint Essential Oil

*Mentha piperita* is a *Lamiaceae* herb native of the Mediterranean region but cultivated worldwide for its uses in cookery and pharmaceutics [48]. Its essential oil, peppermint EO, is rich in menthol and menthone, and possesses antibacterial, antiviral, fungicidal and anti-inflammatory properties [48,83,84,85]. The efficiency of peppermint EO against microorganisms has been evaluated in both liquid and vapour phase, concluding that the concentration of monoterpenes in the oil vapours, such as α-pinene, β-pinene, and limonene, plays a role in inhibiting bacteria growth by inducing extensive damages to the cell membrane [48].

**Figure 3:**
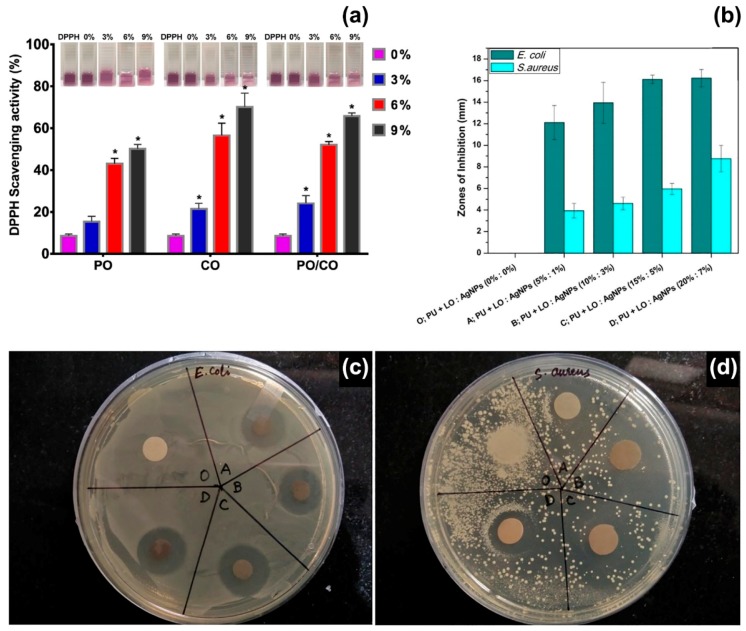
(**a**) 2,2-Diphenyl-1-picryl-hydrazyl-hydrate (DPPH) radical scavenging activity of electrospun fibres containing different concentrations (0%, 3%, 6% and 9% v/v) of peppermint EO (PO), chamomile EO (CO) and their blends (PO/CO). (∗) p < 0.05 versus the control group. Inset: Photographs of DPPH solutions containing the different types of mats. Reproduced from Ref. [50], with permission from the American Chemical Society. (**b**) Zones of inhibition of polyurethane (PU) fibres containing different concentrations of lavender EO (LO) and silver nanoparticles (Ag NPs): sample O: pristine PU fibres; sample A: 5% LO and 1% Ag NPs; sample B: 10% LO and 3% Ag NPs; sample C: 15% LO and 5% Ag NPs; sample D: 20% LO and 7% Ag NPs. Reproduced from Ref. [63] with permission from Elsevier. (**c**,**d**) Photographs of agar plates during disk diffusion assays for *E. coli* and *S. aureus*, respectively. Reproduced from Ref. [63] with permission from Elsevier.

Peppermint EO has been electrospun from PCL [49], cellulose acetate [44], gelatine [50], polyurethane [51], and PEO [52] matrices, and in combination with other bioactive compounds, such as chamomile EO [50], copper sulphate [51], and cerium oxide [52]. Gelatine fibres with an average diameter of ~500 nm were electrospun from acetic acid/water solutions containing 0%, 3%, 6%, and 9% v/v of peppermint EO and 1:1 blends of peppermint and chamomile EOs [50]. The following experimental parameters were used: voltage of 15 kV, flow rate of 0.3 mL/hour and working distance of 10 cm. The fibres containing only peppermint EO (9% v/v) showed the highest inhibitory effect against *E. coli* and *S. aureus*, whereas the addition of chamomile EO enhanced their antioxidant properties. Gelatine fibres with 9% v/v of peppermint EO exhibited a DPPH radical scavenging activity of 50%; while values higher than 60% were collected for fibres with 9% v/v of peppermint/chamomile EOs (Figure 3a), due to the combined action of the two EOs (chamazulen and bisabolene are the main components of chamomile EO) [86]. In another study, the synergistic effect of peppermint EO and copper sulphate was investigated for wound healing applications [51]. Fibres of thermoplastic polyurethane (TPU, medical grade Tecoflex EG 80A) were electrospun from DMF solutions at 8:1 v/v ratio of TPU:peppermint EO, and 8.0:0.5:0.5 v/v ratio of TPU:peppermint EO:CuSO_4_, using a voltage of 11 kV, a flow rate of 0.3 mL/hour and a working distance of 20 cm. Fibres with peppermint EO were characterised by an average diameter of (99 7± 134) nm, while the presence of copper sulphate reduced the size of the fibres to (359 ± 166) nm, possibly due to changes of the electrical conductivity of the polymer solution. Blood coagulation assays were conducted with thromboplastin and prothrombin to assess the anti-coagulant properties of the fibres. The release of the EO determined a 1.2-fold increase of the time needed for blood clot formation, and reduced toxicity toward red blood cells, while no significant effect was reported for copper sulphate. On the other hand, the combined action of peppermint EO and CuSO_4_ increased the viability of Human Dermal Fibroblasts (HDFs), giving cell viability of 144%, compared with 133% and 130% for TPU/peppermint EO and TPU fibres, respectively. The high cytocompatibility was attributed to the small diameter and low hydrophobicity (water contact angle of 82°) of the composite fibres that promoted cell proliferation, while the phenolic components of peppermint EO protected HDFs against oxidative stresses.

Nowadays, electrospinning is regarded as a key technology for the production of biomimetic membranes for tissue engineering and particularly for the treatment of skin wounds [87]. Electrospun mats can be engineered to promote wound healing, while protecting the wound site against bacteria colonisation, releasing drugs at a controlled rate, facilitating the exchange of water and gases, and being mechanically flexible to adapt to the wound region. As in the papers discussed in this section, these features usually result from the combination of organic and inorganic compounds that enhance each other’s bioactivity and, therefore, allow the creation of multifunctional fibres for advanced wound care therapies.

### 3.4. Clove Essential Oil

Clove EO is extracted from the buds, leaves or stems of *Syzygium aromaticum* (synonym: *Eugenia caryophyllata*), a plant of the *Myrtaceae* family that is cultivated in Tanzania, Indonesia, Sri Lanka, and Madagascar [88,89]. This essential oil and its main component, eugenol, are traditionally used as analgesics and antiseptics to treat dental caries and periodontal diseases, being active against cariogenic bacteria, such as *S. mutans* and *S. sobrinus*, and periodontal pathogens, such as *P. gingivalis* and *P. intermedia* [90,91]. They detrimentally impact on the metabolic pathways of other strains of Gram-positive (such as *S. aureus*, *B. cereus*, *B. subtili*) and Gram-negative bacteria (such as *E. coli*, *P. aeruginosa*) by disrupting cell membrane integrity, enhancing membrane wall permeability and therefore leading to bacteria death [92,93].

Clove EO has been encapsulated in electrospun fibres of PCL [53], gelatine [54], PCL/gelatine [55], polyacrylonitrile [56], alginate/PVA [41] and polyvinylpyrrolidone [57], and its efficacy has been investigated against *S. aureus*, *E. coli*, *B. subtilis*, *Klebsiella pneumonia*, *Candida tropicalis*, and *Candida albicans*. PCL/gelatine fibres (with a 7:3 PCL:gelatine ratio) containing different concentrations of clove EO (1.5%, 3.0% and 6.0% v/v) have been produced for wound care applications [55]. The fibres were characterised by an average diameter of 250–300 nm, obtained by electrospinning at a voltage of 20 kV, flow rate of 0.6 mL/hour and working distance of 12 cm. The composite fibres inhibited the growth of *S. aureus* after 6 h of in vitro antibacterial tests, with a reduction of 30–40% of cell viability. However, they were less effective after 24 h, when the bacteria viability returned to values higher than 90%. A similar trend was observed for *E. coli* colonies exposed to electrospun mats containing the lowest concentration of EO: a decrease in cell viability at 6 h, followed by an increase at 24 h. Instead, fibres with 3% and 6% v/v of clove EO induced a gradual decrease of *E. coli* viability, reaching values of 35–40% after 24 h of exposure. Cytotoxicity tests on human dermal fibroblasts demonstrated that PCL-gelatine fibres containing clove EO were biocompatible and did not induce changes in the morphology of the cells, after 48 h. In vitro wound healing (scratch) assays revealed that migration and proliferation of fibroblasts were inhibited in a dose-dependent way, when the cells were exposed to the composite fibres. After 24 h, wound closure rates of ~80% and ~65% were measured for cells exposed to PCL-gelatine fibres with the lowest and highest concentration of EO, respectively. Complete wound closure was instead observed for the control samples (24-well plate) at the same time point.

Previous studies have reported on the dose-dependent cytotoxicity of clove EO [94,95]. When endothelial cells and fibroblasts were incubated in growth media containing different concentrations of clove EO (from 0.002 to 0.250% v/v), it was observed that a concentration as low as 0.03% v/v reduced cell viability to levels lower than 20% after 1 h of exposure [94]. The toxicity was attributed to eugenol, which is expected to induce cell apoptosis (programmed cell death) [95]. It was also demonstrated that eugenol diluted in the cell culture media at concentrations lower than 600 μM caused strong genotoxic effects (DNA damages) on human fibroblasts after 24 h of exposure, because these cells lack metabolic enzymes involved in activation/detoxification processes. The encapsulation of clove EO in electrospun fibres can be a valid strategy to limit the harmful effects of eugenol on cells [96]. By controlling the diffusion rate of eugenol out from the polymeric fibres, it is possible to ensure that the dose released stays within safe limits.

### 3.5. Thyme Essential Oil

Herbaceous perennials and subshrubs of the genus *Thymus* L. (Lamiaceae family) grow in the Mediterranean region, northern Europe, Asia, South America and Australia [97]. Among the *Thymus* species, *Thymus vulgaris* L. (commonly known as thyme) is widely used as aromatic and medicinal plant in food, agriculture, pharmaceutical, and cosmetic industries. Thyme EO possesses strong antibacterial and fungicidal activities, being rich in oxygenated monoterpenes and hydrocarbon monoterpenes: thymol, carvacrol, p-cymene and γ-terpinene [11,98,99,100].

Recently, thyme EO has been encapsulated into fibres of poly(vinylpyrrolidone (PVP) and gelatine (average diameter between 200 and 400 nm) by performing the electrospinning process at a voltage of 25 kV, a flow rate of 0.5 mL/hour and electrodes distance of 17 cm [61]. PVP/gelatine fibrous mats containing 3% w/w of thyme EO were tested against *S. aureus*, *E. coli*, *P. aeruginosa*, and *E. faecalis*, and inhibition zones of 16 mm were recorded for both *S. aureus* and *E. coli*; smaller zones (11–12 cm) were instead measured for the other microorganisms. The encapsulated EO maintained the antibacterial activity even when the electrospun mats were stored at 24 and 37 °C, and inhibition activity against *S. aureus* and *E. coli* was visible after 8 days of incubation. In another study, zein/PEO fibres (average diameter of 6 μm) containing thyme EO (6% w/v) were electrospun from aqueous solutions using a portable and hand-held electrospinning device operating at 15 kV, feeding rate of 30 μL/minute and working distance of 12 cm [58]. The resulting zein/PEO/thyme EO fibres showed antibacterial activity against *E. coli* and *S. aureus*, with inhibition zones in the range of 4–6 cm; whereas fibres without EO were ineffective. Animal tests were performed to assess the healing properties of the zein/PEO/thyme EO fibres when they were deposited in situ on skin wounds. The healing process was accelerated when the animals were treated with zein/PEO/thyme EO dressings, achieving almost complete wound closure after 11 days; differently from wounded animals without dressings that experienced a longer healing time. One of the main advantages of miniaturised and portable electrospinning apparatuses is the deposition of networks of bioactive fibres directly at the wound site to ensure excellent coverage of the injured area [31]. In-situ electrospinning of polymeric solutions containing EOs can better preserve the chemical composition of the therapeutic oils by avoiding the storage of the composite fibres before use. This would limit the evaporation of the most volatile components of the EOs.

### 3.6. Lavender Essential Oil

*Lavandula* species are native to the Mediterranean region but largely distributed and cultivated worldwide as aromatic and medicinal plants [101,102]. The essential oils extracted from *Lavandula angustifolia* and *Lavandula latifoli*a are the most appreciated in pharmaceutical, perfume, and cosmetics industries, aromatherapy and phytotherapy, for their anxiolytic, sedative, anti-inflammatory, antioxidant, and antimicrobial activity [103]. It has been demonstrated that these essential oils and their components impact on the central nervous system and can be used to treat anxiety and reduce stress [102,103]. The main components of *L. angustifolia* and *L. latifolia* EOs are oxygenated monoterpenes: linalool (25–38%), linalyl acetate (25–45%) and lavandulyl acetate (3.4–6.2%) for *L. angustifolia* EO; linalool (34–50%), 1,8-cineole (16–39%) and camphor (10–20%) for *L. latifolia* EO [101]. So far, lavender EOs have been encapsulated in electrospun fibres of sodium alginate [62], polyurethane [63], and polyacrylonitrile [64], to promote wound healing and skin regeneration. Polyurethane (Tecoflex) fibres containing different concentrations of silver nanoparticles (1–7% w/w) and lavender EO (5–20% w/w) have been electrospun at a voltage of 15 kV, flow rate of 0.5 mL/h and needle-to-collector distance of 15 cm [63]. The resulting nanocomposite fibres had a diameter in the range of 0.6–1.0 μm. The fibres containing 15% and 5% w/w of lavender EO and Ag nanoparticles, respectively, were effective against *E. coli* (zone of inhibition of ~16 mm) and *S. aureus* (zone of inhibition of ~6 mm), as shown in Figure 3b,c. Moreover, biocompatibility tests on chicken embryo fibroblasts suggested that these concentrations of lavender EO and silver nanoparticles enhanced cell proliferation, with a 2.4-fold increase in cell viability (6-day incubation) with respect to the control samples (polyurethane fibres without lavender EO and silver nanoparticles). However, cytotoxic effects were recorded for higher concentrations of lavender EO and silver. Previous studies have reported on the toxicity of lavender EO on human foetal lung fibroblasts (MRC-5) [104], human fibroblasts [105], and L929 fibroblasts [106], when the essential oil has been added to the cell culture medium. IC_50_ values of 75 μg/mL have been reported for MRC-5 cells after 48 h of exposure (IC_50_ is the concentration that produces 50% inhibition of cell survival) [104]; while toxic effects have been observed for EO concentrations higher than 12.5 μg/mL administered to L929 fibroblasts for 48 h [106]. The encapsulation of lavender EO into electrospun fibres limits the potential toxicity of the oil that is released at a slow rate, and therefore allows the use of initial EO concentrations up to 200 μg/mL [63,64].

## 4. Conclusions

This review discusses the research conducted so far on the encapsulation of therapeutic essential oils in polymeric electrospun fibres to create functional membranes for biomedical and food packaging applications. The studies in the literature show that the bioactivity of the essential oils is maintained after they are mixed with different polymeric systems and processed by electrospinning. The EOs still possess antimicrobial, anti-inflammatory and antioxidant activity when released from the electrospun mats.

When compared with polymer films, electrospun membranes are characterised by a higher specific surface area (high surface-to-volume ratio of the nanofibres) and microscale porosity (interstitial space between nanofibres). This allows control over the amount and release profile of EOs contained in the electrospun fibres, and therefore limits the cytotoxic effects that some components of EOs have on human cells. One aspect to consider, however, is the possible alteration of the EO chemical composition during fibre formation because of evaporation. For example, chemical analyses have recorded the disappearance of some characteristic bands of peppermint EO when encapsulated in PCL fibres [49], but not in gelatin [50] and cellulose acetate [44] fibres. Similarly, the main components of Manuka EO are retained in electrospun PLA fibres, but this is not the case for tea tree EO in PLA fibres, possibly due to the different evaporation rates of the constituents of the two oils [68].

Future research could investigate the long-term effect of EOs on the physicochemical properties of the polymeric fibres. EOs can work as plasticisers for some polymers, for example PLA, and induce a concentration-dependent reduction of the glass transition temperature of the composite fibres. However, it has not been elucidated yet if EOs can affect the aging of the electrospun mats and induce changes in the fibre’s morphology, mechanical stability, and degradation rate, over time. This could impact on the performances of electrospun fibres containing EOs that are used for biomedical devices and food packaging systems.

## References

[1] Morgan D.J., Okeke I.N., Laxminarayan R., Perencevich E.N., Weisenberg S. (2011). Non-prescription antimicrobial use worldwide: A systematic review. Lancet Infect. Dis..

[2] Zhang W., Mele E. (2018). Phase separation events induce the coexistence of distinct nanofeatures in electrospun fibres of poly (ethyl cyanoacrylate) and polycaprolactone. Mater. Today Commun..

[3] Taiwo A.M. (2019). A review of environmental and health effects of organochlorine pesticide residues in Africa. Chemosphere.

[4] Rani M., Shanker U., Jassal V. (2017). Recent strategies for removal and degradation of persistent and toxic organochlorine pesticides using nanoparticles: A review. J. Environ. Manag..

[5] Carvalho F.P. (2017). Pesticides, environment, and food safety. Food Energy Secur..

[6] Ambrosio C.M.S., de Alencar S.M., de Sousa R.L.M., Moreno A.M., Da Gloria E.M. (2017). Antimicrobial activity of several essential oils on pathogenic and beneficial bacteria. Ind. Crops Prod..

[7] Friedman M. (2015). Antibiotic-resistant bacteria: Prevalence in food and inactivation by food-compatible compounds and plant extracts. J. Agric. Food Chem..

[8] Walia S., Saha S., Tripathi V., Sharma K.K. (2017). Phytochemical biopesticides: Some recent developments. Phytochem. Rev..

[9] Isman M.B., Miresmailli S., Machial C. (2011). Commercial opportunities for pesticides based on plant essential oils in agriculture, industry and consumer products. Phytochem. Rev..

[10] Rehman R., Hanif M.A., Mushtaq Z., Al-Sadi A.M. (2016). Biosynthesis of essential oils in aromatic plants: A review. Food Rev. Int..

[11] Bakkali F., Averbeck S., Averbeck D., Idaomar M. (2008). Biological effects of essential oils—A review. Food Chem. Toxicol..

[12] Sharifi-Rad J., Sureda A., Tenore G.C., Daglia M., Sharifi-Rad M., Valussi M., Tundis R., Sharifi-Rad M., Loizzo M.R., Ademiluyi A.O. (2017). Biological activities of essential oils: From plant chemoecology to traditional healing systems. Molecules.

[13] Pichersky E., Gershenzon J. (2002). The formation and function of plant volatiles: Perfumes for pollinator attraction and defense. Curr. Opin. Plant Biol..

[14] Daviet L., Schalk M. (2010). Biotechnology in plant essential oil production: Progress and perspective in metabolic engineering of the terpene pathway. Flavour Fragr. J..

[15] Biswas K.K., Foster A.J., Aung T., Mahmoud S.S. (2009). Essential oil production: Relationship with abundance of glandular trichomes in aerial surface of plants. Acta Physiol. Plant.

[16] Christianson D.W. (2017). Structural and chemical biology of terpenoid cyclases. Chem. Rev..

[17] Tetali S.D. (2019). Terpenes and isoprenoids: A wealth of compounds for global use. Planta.

[18] McCreath S.B., Delgoda R. (2017). Pharmacognosy: Fundamentals, Applications and Strategies.

[19] Deng Y., Lu S. (2017). Biosynthesis and regulationn of phenylpropanoids in plants. Critical Rev. Plant Sci..

[20] Merino P., Maiuolo L., Delso I., Algieri V., De Nino A., Tejero T. (2017). Chemical approaches to inhibitors of isoprenoid biosynthesis: Targeting farnesyl and geranylgeranyl pyrophosphate synthases. RSC Adv..

[21] Le Roy J., Huss B., Creach A., Hawkins S., Neutelings G. (2016). Glycosylation is a major regulator of phenylpropanoid availability and biological activity in plants. Front. Plant Sci..

[22] Yu O., Jez J.M. (2008). Nature’s assembly line: Biosynthesis of simple phenylpropanoids and polyketides. Plant J..

[23] Sangwan N.S., Farooqi A.H.A., Shabih F., Sangwan R.S. (2001). Regulation of essential oil production in plants. Plant Growth Reg..

[24] Maes C., Bouquillon S., Fauconnier M.-L. (2019). Encapsulation of Essential Oils for the development of biosourced pesticides with controlled release: A review. Molecules.

[25] Prakash B., Kujur A., Yadav A., Kumar A., Singh P.P., Dubey N.K. (2018). Nanoencapsulation: An efficient technology to boost the antimicrobial potential of plant essential oils in food system. Food Control.

[26] Wadhwa G., Kumar S., Chhabra L., Mahant S., Rao R. (2017). Essential oil–cyclodextrin complexes: An updated review. J. Incl. Phenom. Macrocycl. Chem..

[27] Xue J., Wu T., Dai Y., Xia Y. (2019). Electrospinning and electrospun nanofibers: Methods, materials, and applications. Chem. Rev..

[28] Miguel S.P., Figueira D.R., Simões D., Ribeiro M.P., Coutinho P., Ferreira P., Correia I.J. (2018). Electrospun polymeric nanofibres as wound dressings: A review. Coll. Surf. B Biointerfaces.

[29] Soares R.M.D., Siqueira N.M., Prabhakaram M.P., Ramakrishna S. (2018). Electrospinning and electrospray of bio-based and natural polymers for biomaterials development. Mater. Sci. Eng. C.

[30] Han J., Xiong L., Jiang X., Yuan X., Zhao Y., Yang D. (2019). Bio-functional electrospun nanomaterials: From topology design to biological applications. Prog. Polym. Sci..

[31] Mele E. (2016). Electrospinning of natural polymers for advanced wound care: Towards responsive and adaptive dressings. J. Mater. Chem. B.

[32] Thompson C.J., Chase G.G., Yarin A.L., Reneker D.H. (2007). Effects of parameters on nanofiber diameter determined from electrospinning model. Polymer.

[33] Ghobeira R., Asadian M., Vercruysse C., Declercq H., De Geyter N., Morent R. (2018). Wide-ranging diameter scale of random and highly aligned PCL fibers electrospun using controlled working parameters. Polymer.

[34] Rezabeigi E., Demarquette N.R. (2019). Ultraporous membranes electrospun from nonsolvent-induced phase-separated ternary systems. Macromol. Rapid Commun..

[35] Huang C., Thomas N.L. (2018). Fabricating porous poly (lactic acid) fibres via electrospinning. Eur. Polym. J..

[36] Kouparitsas I.K., Mele E., Ronca S. (2019). Synthesis and electrospinning of polycaprolactone from an aluminium-based catalyst: Influence of the ancillary ligand and initiators on catalytic efficiency and fibre structure. Polymers.

[37] Denchai A., Tartarini D., Mele E. (2018). Cellular response to surface morphology: Electrospinning and computational modelling. Front. Bioeng. Biotechnol..

[38] Hajiali H., Contestabile A., Mele E., Athanassiou A. (2018). Influence of topography of nanofibrous scaffolds on functionality of engineered neural tissue. J. Mater. Chem. B.

[39] Wen P., Zhu D.-H., Wu H., Zong M.-H., Jing Y.-R., Han S.-Y. (2016). Encapsulation of cinnamon essential oil in electrospun nanofibrous film for active food packaging. Food Control.

[40] Feng K., Wen P., Yang H., Li N., Lou W.Y., Zong M.H., Wu H. (2017). Enhancement of the antimicrobial activity of cinnamon essential oil-loaded electrospun nanofilm by the incorporation of lysozyme. RSC Adv..

[41] Rafiq M., Hussain T., Abid S., Nazir A., Masood R. (2018). Development of sodium alginate/PVA antibacterial nanofibers by the incorporation of essential oils. Mater. Res. Express.

[42] Wen P., Zhu D.-H., Feng K., Liu F.-J., Lou W.-Y., Li N., Zong M.-H., Wu H. (2016). Fabrication of electrospun polylactic acid nanofilm incorporating cinnamon essential oil/β-cyclodextrin inclusion complex for antimicrobial packaging. Food Chem..

[43] Lin L., Dai Y., Cui H. (2017). Antibacterial poly (ethylene oxide) electrospun nanofibers containing cinnamon essential oil/beta-cyclodextrin proteoliposomes. Carbohydr. Polym..

[44] Liakos I., Rizzello L., Hajiali H., Brunetti V., Carzino R., Pompa P.P., Athanassiou A., Mele E. (2015). Fibrous wound dressings encapsulating essential oils as natural antimicrobial agents. J. Mater. Chem. B.

[45] Figueroa-Lopez K.J., Vicente A.A., Reis M.A., Torres-Giner S., Lagaron J.M. (2019). Antimicrobial and antioxidant performance of various essential oils and natural extracts and their incorporation into biowaste derived poly (3-hydroxybutyrate-co-3-hydroxyvalerate) layers made from electrospun ultrathin fibers. Nanomaterials.

[46] Ardekani-Zadeh A.H., Hosseini S.F. (2019). Electrospun essential oil-doped chitosan/poly (ε-caprolactone) hybrid nanofibrous mats for antimicrobial food biopackaging exploits. Carbohydr. Polym..

[47] Nadeem M., Bhutto M.A., Yu F., Xie X., El-Hamshary H., El-Faham A., Ibrahim U.A., Mo X. (2019). Physico-chemical and biological evaluation of PLCL/SF nanofibers loaded with oregano essential oil. Pharmaceutics.

[48] Tyagi A.K., Malik A. (2011). Antimicrobial potential and chemical composition of *Mentha piperita* oil in liquid and vapour phase against food spoiling microorganisms. Food Control.

[49] Unalan I., Slavik B., Buettner A., Goldmann W.H., Frank G., Boccaccini A.R. (2019). Physical and antibacterial properties of peppermint essential oil loaded poly (ε-caprolactone) (PCL) electrospun fiber mats for wound healing. Front. Bioeng. Biotech..

[50] Tang Y., Zhou Y., Lan X., Huang D., Luo T., Ji J., Mafang Z., Miao X., Wang H., Wang W. (2019). Electrospun gelatin nanofibers encapsulated with peppermint and chamomile essential oils as potential edible packaging. J. Agric. Food Chem..

[51] Jaganathan S.K., Mani M.P., Khudzari A.Z.M. (2019). Electrospun combination of peppermint oil and copper sulphate with conducive physico-chemical properties for wound dressing applications. Polymers.

[52] Bharathi B.S., Stalin T. (2019). Cerium oxide and peppermint oil loaded polyethylene oxide/graphene oxide electrospun nanofibrous mats as antibacterial wound dressings. Mater. Today Commun..

[53] Sahal G., Nasseri B., Ebrahimi A., Bilkay I.S. (2019). Electrospun essential oil-polycaprolactone nanofibers as antibiofilm surfaces against clinical Candida tropicalis isolates. Biotechnol. Lett..

[54] Cui H., Bai M., Rashed M.M., Lin L. (2018). The antibacterial activity of clove oil/chitosan nanoparticles embedded gelatin nanofibers against *Escherichia coli* O157: H7 biofilms on cucumber. Int. J. Food Microbiol..

[55] Unalan I., Endlein S.J., Slavik B., Buettner A., Goldmann W.H., Detsch R., Boccaccini A.R. (2019). Evaluation of electrospun poly (ε-caprolactone)/gelatin nanofiber mats containing clove essential oil for antibacterial wound dressing. Pharmaceutics.

[56] Yadav R., Balasubramanian K. (2014). Polyacrylonitrile/Syzygium aromaticum hierarchical hydrophilic nanocomposite as a carrier for antibacterial drug delivery systems. RSC Adv..

[57] Tonglairoum P., Ngawhirunpat T., Rojanarata T., Kaomongkolgit R., Opanasopit P. (2016). Fabrication and evaluation of nanostructured herbal oil/hydroxypropyl-β-cyclodextrin/polyvinylpyrrolidone mats for denture stomatitis prevention and treatment. AAPS PharmSciTech.

[58] Liu J.X., Dong W.H., Mou X.J., Liu G.S., Huang X.W., Yan X., Zhou C.F., Jiang S., Long Y.Z. (2020). In situ electrospun zein/thyme essential oil-based membranes as an effective antibacterial wound dressing. ACS Appl. Bio Mater..

[59] Dadras Chomachayi M., Solouk A., Akbari S., Sadeghi D., Mirahmadi F., Mirzadeh H. (2018). Electrospun nanofibers comprising of silk fibroin/gelatin for drug delivery applications: Thyme essential oil and doxycycline monohydrate release study. J. Biomed. Mater. Res. Part A.

[60] Lin L., Liao X., Cui H. (2019). Cold plasma treated thyme essential oil/silk fibroin nanofibers against Salmonella Typhimurium in poultry meat. Food Packag. Shelf Life.

[61] Çallıoğlu F.C., Güler H.K., Çetin E.S. (2019). Emulsion electrospinning of bicomponent poly (vinyl pyrrolidone)/gelatin nanofibers with thyme essential oil. Mater. Res. Express.

[62] Hajiali H., Summa M., Russo D., Armirotti A., Brunetti V., Bertorelli R., Athanassiou A., Mele E. (2016). Alginate-lavender nanofibers with antibacterial and anti-inflammatory activity to effectively promote burn healing. J. Mater. Chem. B.

[63] Sofi H.S., Akram T., Tamboli A.H., Majeed A., Shabir N., Sheikh F.A. (2019). Novel lavender oil and silver nanoparticles simultaneously loaded onto polyurethane nanofibers for wound-healing applications. Int. J. Pharm..

[64] Balasubramanian K., Kodam K.M. (2014). Encapsulation of therapeutic lavender oil in an electrolyte assisted polyacrylonitrile nanofibres for antibacterial applications. RSC Adv..

[65] Antunes M.D., da Silva Dannenberg G., Fiorentini Â.M., Pinto V.Z., Lim L.T., da Rosa Zavareze E., Dias A.R.G. (2017). Antimicrobial electrospun ultrafine fibers from zein containing eucalyptus essential oil/cyclodextrin inclusion complex. Int. J. Biolog. Macromol..

[66] Da Silva F.T., da Cunha K.F., Fonseca L.M., Antunes M.D., El Halal S.L.M., Fiorentini Â.M., da Rosa Zavareze E., Dias A.R.G. (2018). Action of ginger essential oil (*Zingiber officinale*) encapsulated in proteins ultrafine fibers on the antimicrobial control in situ. Int. J. Biolog. Macromol..

[67] Cui H., Bai M., Lin L. (2018). Plasma-treated poly (ethylene oxide) nanofibers containing tea tree oil/beta-cyclodextrin inclusion complex for antibacterial packaging. Carbohydr. Polym..

[68] Zhang W., Huang C., Kusmartseva O., Thomas N.L., Mele E. (2017). Electrospinning of polylactic acid fibres containing tea tree and manuka oil. React. Funct. Polym..

[69] Wang P., Mele E. (2018). Effect of antibacterial plant extracts on the morphology of electrospun poly (lactic acid) fibres. Materials.

[70] D’Agostino M., Tesse N., Frippiat J.P., Machouart M., Debourgogne A. (2019). Essential oils and their natural active compounds presenting antifungal properties. Molecules.

[71] Zhang Y., Liu X., Wang Y., Jiang P., Quek S. (2016). Antibacterial activity and mechanism of cinnamon essential oil against *Escherichia coli* and *Staphylococcus aureus*. Food Control.

[72] Huang D.F., Xu J.G., Liu J.X., Zhang H., Hu Q.P. (2014). Chemical constituents, antibacterial activity and mechanism of action of the essential oil from *Cinnamomum cassia* bark against four food-related bacteria. Microbiology.

[73] Wang Y., Zhang Y., Shi Y.Q., Pan X.H., Lu Y.H., Cao P. (2018). Antibacterial effects of cinnamon (*Cinnamomum zeylanicum*) bark essential oil on Porphyromonas gingivalis. Microb. Pathogen..

[74] Goni P., López P., Sánchez C., Gómez-Lus R., Becerril R., Nerín C. (2009). Antimicrobial activity in the vapour phase of a combination of cinnamon and clove essential oils. Food Chem..

[75] Kfoury M., Landy D., Fourmentin S. (2018). Characterization of cyclodextrin/volatile inclusion complexes: A Review. Molecules.

[76] Topuz F., Uyar T. (2019). Electrospinning of cyclodextrin functional nanofibers for drug delivery applications. Pharmaceutics.

[77] Han J.-W., Ruiz-Garcia L., Qian J.-P., Yang X.-T. (2018). Food packaging: A comprehensive review and future trends. Compreh. Rev. Food Sci. Food Saf..

[78] Majed R., Faille C., Kallassy M., Gohar M. (2016). Bacillus cereus biofilms—Same, only different. Front. Microbiol..

[79] Cui H., Zhang C., Li C., Lin L. (2019). Antibacterial mechanism of oregano essential oil. Ind. Crops Prod..

[80] Pezzani R., Vitalini S., Iriti M. (2017). Bioactivities of *Origanum vulgare* L.: An update. Phytochem. Rev..

[81] Lv F., Liang H., Yuan Q., Li C. (2011). In vitro antimicrobial effects and mechanism of action of selected plant essential oil combinations against four food-related microorganisms. Food Res. Int..

[82] Lu M., Dai T., Murray C.K., Wu M.X. (2018). Bactericidal property of oregano oil against multidrug-resistant clinical isolates. Front. Microbiol..

[83] Soković M., Vukojević J., Marin P., Brkić D., Vajs V., Van Griensven L. (2009). Chemical composition of essential oils of thymus and mentha species and their antifungal activities. Molecules.

[84] İşcan G., Kïrïmer N., Kürkcüoǧlu M., Başer H.C., Demirci F. (2002). Antimicrobial screening of *Mentha piperita* essential oils. J. Agric. Food Chem..

[85] Sun Z., Wang H., Wang J., Zhou L., Yang P. (2014). Chemical composition and anti-inflammatory, cytotoxic and antioxidant activities of essential oil from leaves of *Mentha piperita* grown in China. PloS ONE.

[86] Sizova N.V. (2012). Composition and antioxidant activity of essential oils containing azulene derivatives. Pharm. Chem. J..

[87] Fahimirad S., Ajalloueian F. (2019). Naturally-derived electrospun wound dressings for target delivery of bio-active agents. Int. J. Pharm..

[88] Miyazawa M., Hisama M. (2003). Antimutagenic activity of phenylpropanoids from clove (*Syzygium aromaticum*). J. Agric. Food Chem..

[89] Fichi G., Flamini G., Giovanelli F., Otranto D., Perrucci S. (2007). Efficacy of an essential oil of *Eugenia caryophyllata* against *Psoroptes cuniculi*. Exp. Parasitol..

[90] Chaieb K., Hajlaoui H., Zmantar T., Kahla-Nakbi A.B., Rouabhia M., Mahdouani K., Bakhrouf A. (2007). The chemical composition and biological activity of clove essential oil, *Eugenia caryophyllata* (*Syzigium aromaticum* L. Myrtaceae): A short review. Phytoth. Res..

[91] Moon S.E., Kim H.Y., Cha J.D. (2011). Synergistic effect between clove oil and its major compounds and antibiotics against oral bacteria. Arch. Oral Biol..

[92] Khalil A.A., ur Rahman U., Khan M.R., Sahar A., Mehmood T., Khan M. (2017). Essential oil eugenol: Sources, extraction techniques and nutraceutical perspectives. RSC Adv..

[93] Xu J.G., Liu T., Hu Q.P., Cao X.M. (2016). Chemical composition, antibacterial properties and mechanism of action of essential oil from clove buds against *Staphylococcus aureus*. Molecules.

[94] Prashar A., Locke I.C., Evans C.S. (2006). Cytotoxicity of clove (*Syzygium aromaticum*) oil and its major components to human skin cells. Cell Prolif..

[95] Slameňová D., Horváthová E., Wsólová L., Šramková M., Navarová J. (2009). Investigation of anti-oxidative, cytotoxic, DNA-damaging and DNA-protective effects of plant volatiles eugenol and borneol in human-derived HepG2, Caco-2 and VH10 cell lines. Mutat. Res. Genet. Toxicol. Environ. Mutagen..

[96] Li Z., Zhou P., Zhou F., Zhao Y., Ren L., Yuan X. (2018). Antimicrobial eugenol-loaded electrospun membranes of poly (ε-caprolactone)/gelatin incorporated with REDV for vascular graft applications. Coll. Surf. B Biointerfaces.

[97] Nabavi S.M., Marchese A., Izadi M., Curti V., Daglia M., Nabavi S.F. (2015). Plants belonging to the genus Thymus as antibacterial agents: From farm to pharmacy. Food Chem..

[98] Tariq S., Wani S., Rasool W., Bhat M.A., Prabhakar A., Shalla A.H., Rather M.A. (2019). A comprehensive review of the antibacterial, antifungal and antiviral potential of essential oils and their chemical constituents against drug-resistant microbial pathogens. Microb. Pathogen..

[99] Pirbalouti A.G., Hashemi M., Ghahfarokhi F.T. (2013). Essential oil and chemical compositions of wild and cultivated *Thymus daenensis* Celak and *Thymus vulgaris* L.. Ind. Crops Prod..

[100] Rota M.C., Herrera A., Martínez R.M., Sotomayor J.A., Jordán M.J. (2008). Antimicrobial activity and chemical composition of *Thymus vulgaris*, *Thymus zygis* and *Thymus hyemalis* essential oils. Food Control.

[101] Aprotosoaie A.C., Gille E., Trifan A., Luca V.S., Miron A. (2017). Essential oils of *Lavandula genus*: A systematic review of their chemistry. Phytochem. Rev..

[102] Dobetsberger C., Buchbauer G. (2011). Actions of essential oils on the central nervous system: An updated review. Flavour Fragr. J..

[103] Cavanagh H.M.A., Wilkinson J.M. (2002). Biological activities of lavender essential oil. Phytotherapy Res..

[104] Nikolić M., Jovanović K.K., Marković T., Marković D., Gligorijević N., Radulović S., Soković M. (2014). Chemical composition, antimicrobial, and cytotoxic properties of five Lamiaceae essential oils. Ind. Crops Prod..

[105] Prashar A., Locke I.C., Evans C.S. (2004). Cytotoxicity of lavender oil and its major components to human skin cells. Cell Prolif..

[106] Gavanji S., Mohammadi E., Larki B., Bakhtari A. (2014). Antimicrobial and cytotoxic evaluation of some herbal essential oils in comparison with common antibiotics in bioassay condition. Integr. Med. Res..

